# Survival rate of cervical cancer in Asian countries: a systematic review and meta-analysis

**DOI:** 10.1186/s12905-023-02829-8

**Published:** 2023-12-14

**Authors:** Mohebat Vali, Zahra Maleki, Hossein-Ali Nikbakht, Soheil Hassanipour, Aida Kouhi, Sina Nazemi, Maryam hajizade-valokolaee, MohammadReza Nayeb, Haleh Ghaem

**Affiliations:** 1 Student Research Committee, Shiraz University of Medical Sciences, Shiraz, Iran; 2 Social Determinants of Health Research Center, Health Research Institute, Department of Biostatistics & Epidemiology, School of Public Health, Babol University of Medical Sciences, Babol, Iran; 3 Gastrointestinal and Liver Diseases Research Center, Guilan University of Medical Sciences, Rasht, Iran; 4 Department of Pathology, Keck School of Medicine, University of Southern California, Los Angeles, CA USA; 5 Department of Radiology, Keck School of Medicine, University of Southern California, Los Angeles, CA USA; 6 Department of Health, Health Systems Research, Health Research Institute, Babol University of Medical Sciences, Babol, Iran; 7 Non-Communicable Diseases Research Center, Shiraz University of Medical Sciences, Shiraz, Iran; 8 Department of Epidemiology, School of Health, Shiraz University of Medical Sciences, Shiraz, Iran

**Keywords:** Survival rate, Cervical cancer, Asian countries, Meta analysis

## Abstract

**Objective:**

Cancer is one of the main causes of death, and cervical cancer is the fourth most common cancer and the fourth leading cause of death from malignancy among women. Knowing the survival rate is used to evaluate the success of current treatments and care. This study was conducted to assess the survival rate of cervical cancer in Asia.

**Methods:**

This systematic survey was conducted on four international databases, including Medline/PubMed, ProQuest, Scopus, and Web of Knowledge, and includes manuscripts that were published until the end of August 2021. Selected keywords were searched for international databases including cervical neoplasms [mesh], survival analysis or survival or survival rate, Asian countries (name of countries). The Newcastle-Ottawa Qualitative Evaluation Form was used for cohort studies to evaluate the quality of the articles. The analysis process was performed to evaluate the heterogeneity of the studies using the Cochran test and I^2^ statistics. Additionally, a meta-regression analysis was performed based on the year of the study.

**Results:**

A total of 1956 articles were selected and reviewed based on their title. The results showed that 110 articles met the inclusion criteria. According to the randomized model, the 1, 3, 5, and 10-year survival rates of cervical cancer were 76.62% (95% Confidence Interval (CI), 72.91_80.34), 68.77% (95% CI, 64.32_73.21), 62.34% (95% CI, 58.10_66.59), and 61.60% (95% CI, 52.31_70.89), respectively. Additionally, based on the results of meta-regression analysis, there was an association between the year of the study and the survival rate, elucidating that the survival rate of cervical cancer has increased over the years.

**Conclusions:**

Results can provide the basic information needed for effective policy making, and development of public health programs for prevention, diagnosis, and treatment of cervical cancer.

**Supplementary Information:**

The online version contains supplementary material available at 10.1186/s12905-023-02829-8.

## Introduction

In the 21st century, cancer is one of the leading causes of death, and cervical cancer is the fourth most common cancer and the fourth leading cause of death from malignancy in women. It is the first and most common gynecological malignancy in parts of sub-Saharan Africa, Melanesia, East Africa (Malawi), South America, and South-Eastern Asia [1]. Moreover, it is the second most common cancer among women after breast cancer in developing countries [2]. According to the World Health Organization, 85% of cervical cancers occur in developing countries. Every year, about 500,000 women suffer from this malignancy globally, and the mortality rate is 200,000 [3, 4].

Given that many cervical cancers are caused by human papillomavirus 16 and 18, which account for 70% of all cervical cancers worldwide, a large proportion of cervical cancers can be prevented by screening the cervix for presence of the virus [5]. While western countries have introduced human papillomavirus vaccination programs and have effective screening strategies, low- and middle-income countries lack organized screening and vaccination programs, which often results in late-stage diagnosis of the disease in the latter regions. Due to the long period before the invasion, with an appropriate screening program and effective treatment of primary lesions, the development of this cancer can be prevented in the early stages [6]. Although cervical cancer has not yet been eliminated despite these strategies, the incidence has been reduced, and since cases are diagnosed earlier, the survival of patients has improved. In Asia, the lowest and highest standardized age-related incidences of cervical cancer are in China and Thailand, at 3.2 and 23.8 per 100,000, respectively [7, 8]. Also, a study by Chen et al. in China showed that the one-year, three-year, and five-year survival rates of cervical cancer are 60.3%, 44.3%, and 34.3%, respectively, and the results of sensitivity analysis showed that early treatment is correlated to long-term survival [9]. According to a study conducted in Iran in 2022, the five-year survival rate of cervical cancer was reported to be 35.5%, which was higher than the survival rate of other types of cancers in women [10]. Studies on the survival of patients with cervical cancer in different cultural, racial, and genetic populations have shown that these factors will make a difference in multi-year survival in these different population groups. Since no comprehensive study has been conducted in Asia to assess the multi-year survival rate of patients with cervical cancer, we conducted this study to perform a systematic review and meta-analysis to determine the survival rate in Asian countries. This information can help in development of effective public health interventions for prevention, diagnosis, and treatment of cervical cancer.

## Methods

The present study is a systematic review and meta-analysis of cervical cancer survival rates. This study was designed and implemented in 2021. The reporting method for the present study is based on the PRISMA (Preferred Reporting Items for Systematic Reviews and Meta-Analysis) checklist [11].

### Search strategy (methodology)

Researchers in the study surveyed four international databases: Medline/PubMed, ProQuest, Scopus, and the Web of Knowledge, for articles that were published by August 2021. Google Scholar for gray literature was also searched.

Selected keywords for international databases included: Uterine Cervical Neoplasms [Mesh], Survival OR Survival Analysis OR Survival Rate, Asian Countries (Names of countries) ( Appendix 1(.

The collected data was entered into EndNote X7 software, and duplicated content (articles) was automatically deleted. It should be mentioned that two researchers examined the articles separately. The search strategy is presented in Appendix 1.

### Inclusion and exclusion criteria

All observational studies (cross-sectional, case-control, and cohort) that were published until the end of August 2021, and in which the observed survival of cervical cancer was mentioned and published in English, were included in the study without any time limit.

Review studies and meta-analyses were excluded. Also, studies that did not say how many people were in the sample or did not give a confidence interval for the survival rate were not included in the meta-analysis.

### Quality assessment (evaluation)

The Newcastle-Ottawa quality assessment scale was used to evaluate articles’ quality. This tool includes three parts: (1) Selection (four questions); (2) Comparison (one question); and (3) Result (three questions). Based on the final score, the quality of the articles is divided into the following three categories: Good (three or four stars in the selection section, one or two stars in the comparison section, and two or three stars in the results section); Average (two stars in the selection section, one or two stars in the comparison section, and two or three stars in the results section); and Poor (zero or one star in the selection section, zero stars in the comparison section, and zero or one star in the result section) [12].

### Screening of studies

Two researchers conducted the initial search for studies. In addition to screening the quality of studies, extraction of data and evaluation of the quality of articles were performed independently by two researchers. In the absence of consensus between the two researchers, the team leader made the final decision about that article.

### Data extraction form

The data from the final articles that were included in the study process were extracted (by a previously prepared checklist). This checklist included the authors’ name, the publication year, the length of the study, sample size, cancer type, country, and 1-, 3-, 5-, and 10-year survival rates.

### Statistical analysis

Heterogeneity between studies was evaluated using the Cochran test (at a significance level of less than 0.1) combined with the I^2^ statistic. In the case of heterogeneity, the random effects model was used with the inverse variance method, and in the case of no heterogeneity, the fixed effects model was used. In the case of heterogeneity among studies, methods such as meta-regression and subgroup analysis were used. All analyses were performed using STATA version 16 and MEDCALC version 14 statistical software.

### Risk of bias among studies

A random-effects model was used to reduce the risk of bias in studies [13, 14]. Egger’s test for publication bias was also used to evaluate the risk of publication bias [15].

## Results

### Study selection

**Fig. 1:**
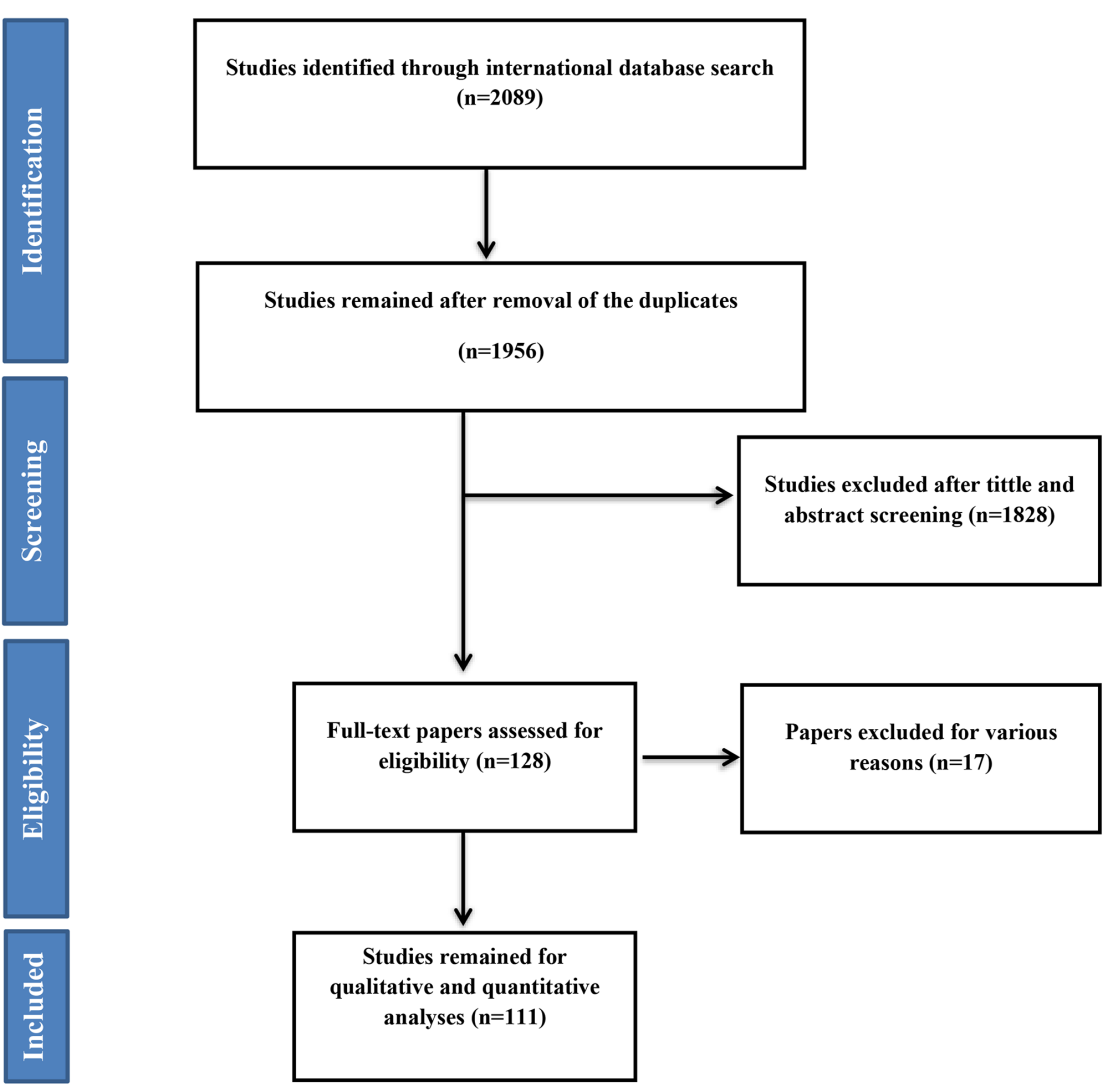
Flowchart of the included eligible studies in systematic review

A total of 2089 articles were found, and after removing duplicated articles, 1956 articles were reviewed in terms of title and abstract. After thoroughly assessing the articles, 128 articles entered the next phase, in which the full texts were examined, and 110 articles(289 records) were selected for the final analysis. It should be noted that the references of the final articles were also reviewed to add relevant studies. The process of selecting studies is presented in Fig. 1.

### Study characteristics

Studies that were included in the analysis were published between 1990 and 2021. Of the studies that were initially found, 110(289 records) were eligible in this period and reported the survival of cervical cancer patients in Asian countries. Fifty-one reports were from China, 23from Hong Kong, 42 from India, 4 from Iran, 29 from Japan, 29 from South Korea, 4 from Kuwait, 7 from Malaysia, 15 from the Philippines, 15 from Saudi Arabia, 15 from Singapore, 27 from Taiwan, 12 from Thailand, 7 from Turkey, and 12 from Vietnam. Descriptive information about these studies is given in Appendix 2.

### Quality appraisal

According to the assessment using the Newcastle-Ottawa scale, 80 studies were of Good quality, and 30 articles were of Average quality. The articles’ quality assessment results are shown in Appendix 3.

### Heterogeneity

The results of the Chi-squared test and I^2^ index showed significant heterogeneity between studies. In the survival analysis for cervical cancer in Asian countries, the one-year survival rate was I^2^ = 99.31%, *P* < 0.001, for three-year survival rate was I^2^ = 98.96%, *P* < 0.001, for five-year survival rate was I^2^ = 98.95%, *P* < 0.001, and for ten-year survival rate was I^2^ = 99.15%, *P* < 0.001. The random-effects model was used to achieve the results for all analyses.

### Results of the meta-analysis

First, the articles were sorted according to the publication year of the study, and then the obtained survival rates were categorized into one, three, five, and ten-year survival rates. Meta-regression was also performed based on the year of the study.

### One-year survival rate of cervical cancer in Asian countries

**Fig. 2:**
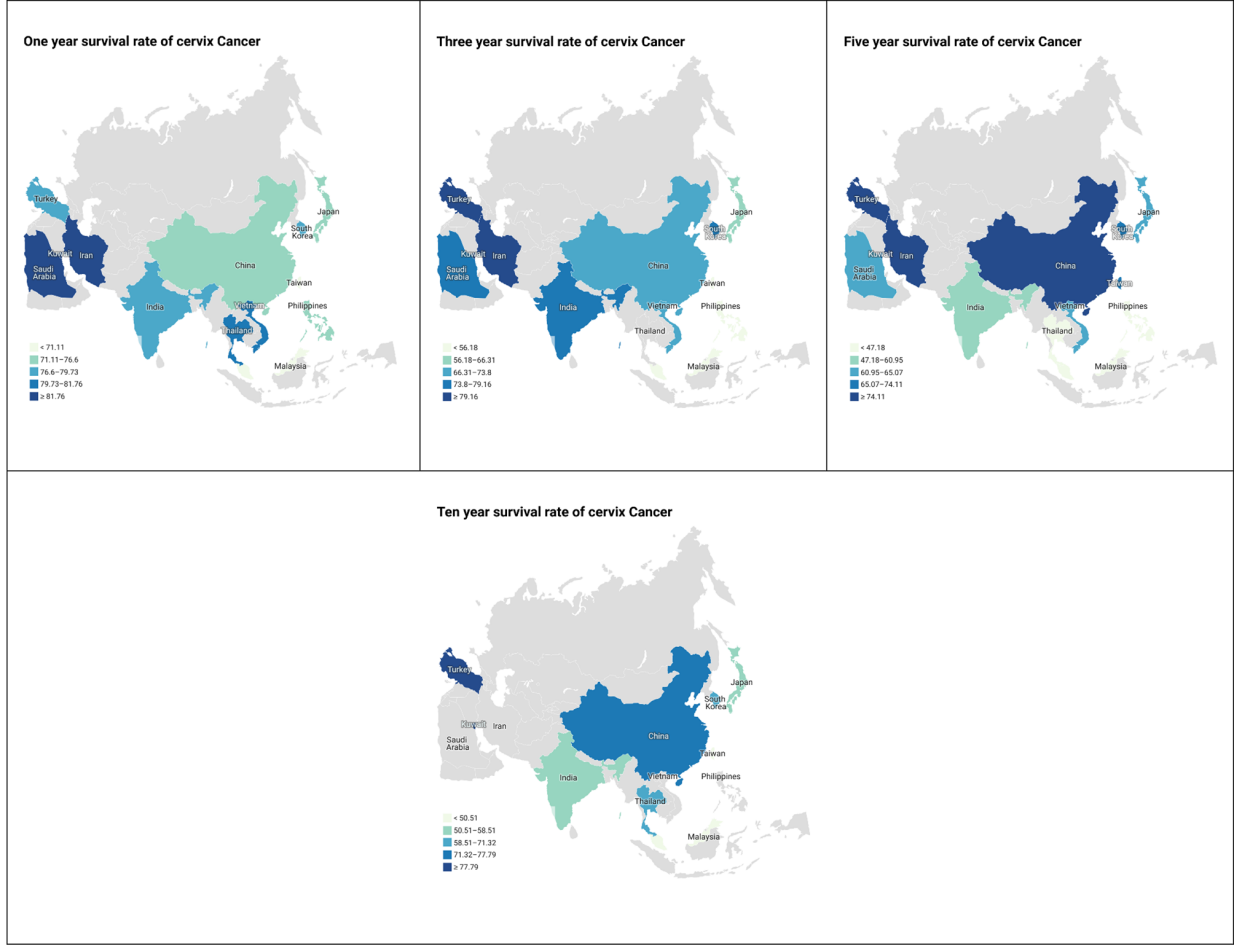
One, three, five, and ten year survival of survival rate of cervix Cancer in Asian Countries

From 110 final articles(289 records) that were included in the analyses, 98 studies reported one-year survival of patients. Based on the random-effect model, the study results showed that the one-year survival was 76.62% (95% Confidence Interval (CI), 72.91–80.34). (Table 1; Fig. 2).

**Table 1:** Result of meta-analysis and heterogeneity of survival rate of cervix Cancer in Asian Countries base on each country and year of survival

Country	Total	Year of Survival															
		1				3				5				10			
		N	Effect estimate	I^2^	P	N	Effect estimate	I^2^	P	N	Effect estimate	I^2^	P	N	Effect estimate	I^2^	P
China	51	15	74.84 (65.45, 84.23)	98.03	≤ 0.001	16	71.97 (61.85, 82.09)	99.20	≤ 0.001	18	75.36 (67.04, 83.67)	99.20	≤ 0.001	2	76.08 (40.47, 111.69)	88.61	0.003
Hong Kong	23	7	81.51 (67.29, 95.72)	99.70	≤ 0.001	7	65.71 (46.23, 85.19)	99.66	≤ 0.001	8	59.01 (41.99, 76.03)	99.45	≤ 0.001	1	73.20 (68.62, 77.77)	-	-
India	42	16	77.77 (69.45, 86.10)	97.64	≤ 0.001	10	76.12 (67.05, 85.20)	95.63	≤ 0.001	13	59.85 (50.18, 69.51)	96.22	≤ 0.001	3	51.99 (44.84, 59.13)	68.35	0.035
Iran	4	2	89.98 (71.95, 108.00)	97.08	≤ 0.001	1	83.10 (77.73, 88.46)	-	-	1	78.20 (68.94, 87.45)	-	-	-	-	-	-
Japan	29	9	74.07 (65.85, 82.28)	97.07	≤ 0.001	6	64.78 (54.07, 75.48)	93.70	≤ 0.001	8	64.29 (46.21, 82.38)	98.84	≤ 0.001	6	55.09 (32.20, 77.98)	99.59	≤ 0.001
Korea	29	8	79.53 (69.24, 89.83)	98.92	≤ 0.001	8	74.26 (59.72, 88.79)	99.11	≤ 0.001	8	66.15 (49.53, 82.77)	99.34	≤ 0.001	5	60.79 (34.35, 87.24)	99.64	≤ 0.001
Kuwait	4	1	86.30 (80.76, 91.83)	-	-	1	84.40 ( 78.58, 90.21)	-	-	1	73.80 (66.77, 80.82)	-	-	1	88.70 ( 83.59, 93.80)	-	-
Malaysia	7	1	35.20 (26.34, 44.05)	-	-	1	41.20 (32.08, 50.31)	-	-	4	38.21 (23.37, 53.06)	91.57	≤ 0.001	1	44.60 (35.38, 53.81)	-	-
Philippines	15	3	71.73 (63.82, 79.64)	88.41	≤ 0.001	6	51.17 (37.95, 64.39)	95.42	≤ 0.001	6	36.16 (21.22, 51.09)	95.54	≤ 0.001	-	-	-	-
Saudi Arabia	15	4	82.76 (74.55, 90.98)	83.28	≤ 0.001	5	76.54 (65.41, 87.67)	90.75	≤ 0.001	6	65.01 (55.49, 74.52)	86.11	≤ 0.001	-	-	-	-
Singapore	15	4	68.63 (58.38, 78.88)	98.18	≤ 0.001	4	59.52 (55.11, 63.92)	88.22	≤ 0.001	4	49.42 (42.01, 56.84)	95.74	≤ 0.001	-	-	-	-
Taiwan	27	10	65.19 (43.91, 86.47)	99.92	≤ 0.001	5	44.18 (27.17, 61.20)	97.77	≤ 0.001	11	65.17 (56.65, 73.68)	96.99	≤ 0.001	1	26.10 (21.50, 30.69)	-	-
Thailand	12	7	80.77 (69.52, 92.02)	98.83	≤ 0.001	-	-	-	-	1	15.20 (9.60, 20.79)	-	-	4	70.06 (53.43, 86.70)	97.21	≤ 0.001
Turkey	7	1	79.66 (67.81, 91.50)	-	-	2	96.46 (92.81, 100.11)	0.00	0.663	3	78.97 (71.58, 86.36)	55.31	0.109	1	84.62 (73.64, 95.59)	-	-
Vietnam	12	5	79.83 (65.82, 93.84)	98.39	≤ 0.001	4	68.73 (59.02, 78.44)	91.92	≤ 0.001	3	61.68 (44.62, 78.74)	95.40	≤ 0.001	-	-	-	-
**Overall**	289	93	76.62 (72.91, 80.34)	99.31	≤ 0.001	76	68.77 (64.32, 73.21)	98.96	≤ 0.001	95	62.34 (58.10, 66.59)	98.95	≤ 0.001	25	61.60 (52.31, 70.89)	99.15	≤ 0.001

*NR; Not reported

### Three-year survival rate of cervical cancer in Asian countries

From 110final articles (289 records) that were included in the analyses, 78 studies reported three-year survival of patients. Based on the random-effect model, the results of the study showed that the three-year survival was 68.77% (95% CI, 64.32–73.21). (Table 1; Fig. 2).

### Five-year survival rate of cervical cancer in Asian countries

From 110final articles(289 records) that were included in the analyses, 96 studies reported the five-year survival of patients. Based on the random-effect model, the results of the study showed that the five-year survival was 62.34% (95% CI, 58.10-66.59). (Table 1; Fig. 2).

### Ten-year survival rate of cervical cancer in Asian countries

From 110 final articles(289 records) that were included in the analyses, 25 studies reported the ten-year survival of patients. Based on the random-effect model, the results of the study showed that the ten-year survival was 61.60% (95% CI, 52.31–70.89). (Table 1; Fig. 2).

### Cervical cancer survival rate in each Asian country

Overall, survival rates of cervical cancer in 15 countries are depicted in Table 1; Fig. 2. The highest one-, three-, five-, and ten-year survival rates were reported in Iran (89.98), Turkey (96.46), Turkey (78.97), and Kuwait (88.70), respectively. The lowest one-, three-, five-, and ten-year survival rates were reported in Malaysia (35.20), Malaysia (41.20), Thailand (15.20), and Taiwan (26.10), respectively.

### Meta-regression of cervical cancer survival rate in Asian countries

**Fig. 3:**
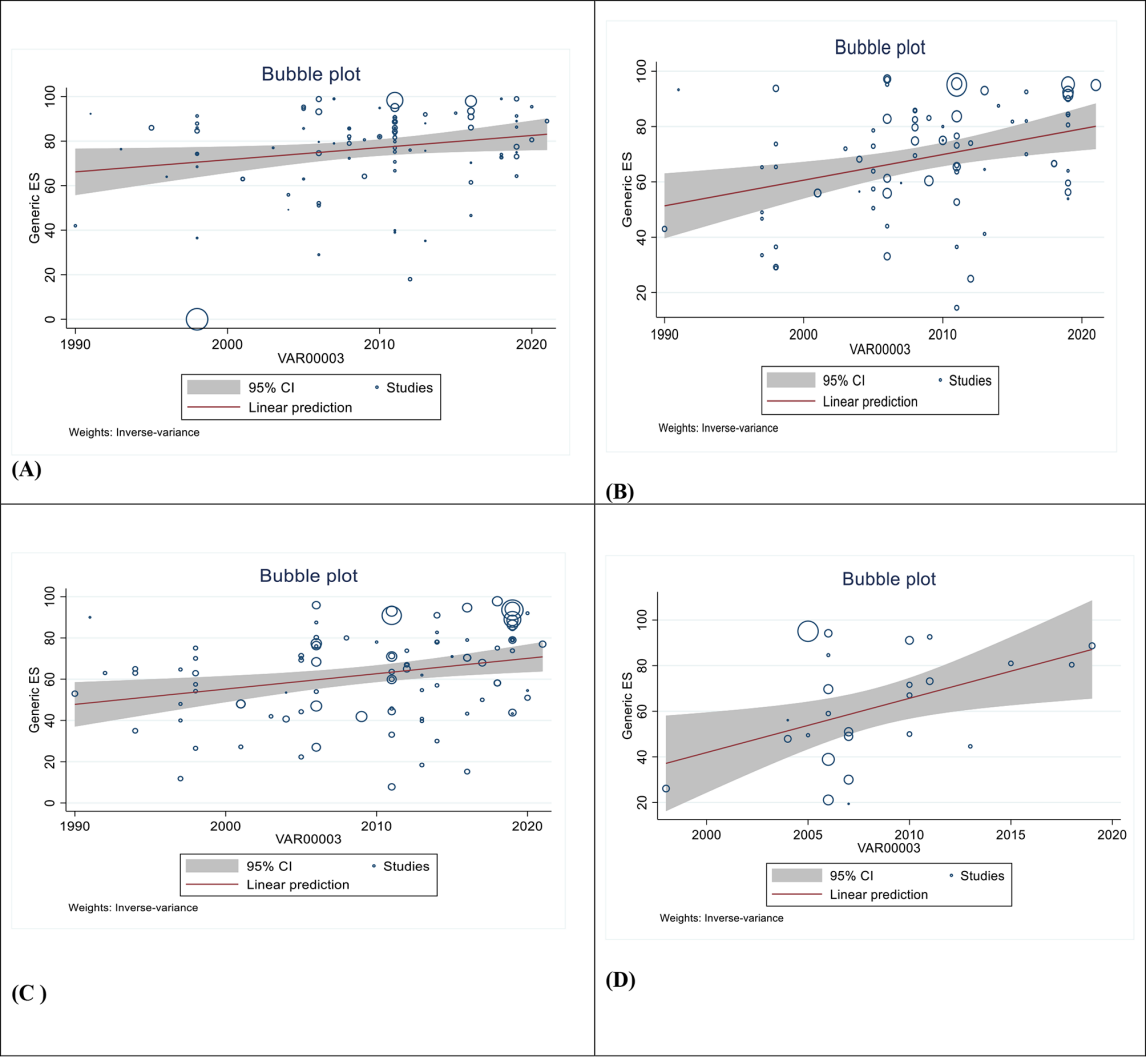
Bubble plot of standard error by point estimate for assessment of meta regression (1,3 and 5 year Cervical cancer survival rate) [**A**: One Cervical cancer survival rate, **B**: three Cervical cancer survival rate, **C**: Five Cervical cancer survival rate, **D**: Ten Cervical cancer survival rate]

There was a correlation between the study year and the survival rate. Survival rates for one year (Reg Coef = 0.5439, *p* = 0.037), three years (Reg Coef = 0.9275, *p* = 0.002), five years (Reg Coef = 0.7402, *p* = 0.004), and ten years (Reg Coef = 2.38, *p* = 0.012) have increased in recent years, and this increase in survival was statistically significant (Fig. 3).

### Publication bias

We drew Funnel plots in order to investigate the publication bias for one, three, five- and ten-year cervical cancer survival in Asian countries. Egger test results confirmed this bias.

(One-year bias: -0.91, 95% CI = -2.76 to 0.93; *P* = 0.334).

(Three-year bias: -1.11, 95% CI = -3.08 to 0.86; *P* = 0.272).

(Five-year bias: -1.03, 95% CI = -3.25 to 1.19; *P* = 0.365).

(Ten-year bias: -1.74, 95% CI = -5.44 to 1.96; *P* = 0.357). (Appendix 4).

## Discussion

Cervical cancer is a highly preventable cancer, yet it is annually responsible for over 300,000 deaths worldwide [16]. The present study examined the cervical cancer survival rate at 1, 3, 5, and 10 years. According to the results, the one-year survival rate is 76.62%, the three-year rate is 68.77%, the five-year rate is 62.34%, and the ten-year rate is 61.60%). According to this study, the survival rate is improving, and was reported to be significantly higher for recent years. Based on results, Taiwan had the lowest ten-year survival rate, while Kuwait had the highest survival rate.

The results of the study showed that the survival rate of cervical cancer patients decreases if the cancer was diagnosed in the late stage of the disease and in fact, among the most important factors related to the survival rate of patients with cervical cancer is the stage of the disease. As seen, the survival of Cervical Cancer in Asia is not much different between 1, 3, 5 and 10 year survival. Perhaps one of the reasons is its timely identification in this geographical area, which has caused the survival of 1, 3, 5, and 10 years of this cancer to be seen with the use of timely screening. Of course, the up-to-date treatment methods should not be neglected, and perhaps another reason for this observation is the progress of the treatment of this cancer and finally the survival of 1, 3, 5 and 10 is close to each other. In a study in India, the survival rates for stage I, stage II, stage III, and stage IV were reported to be 83.5%, 80.6%, 66.0%, and 37.1%, respectively, and in this study, stage of disease was the only significant prognostic factor mentioned for survival [17]. Lack of information about cervical cancer and Pap smear screening, lack of time and financial resources, fear of medical intervention, and unwillingness to participate are among the most preventing factors for screening in India [18]. Patients with cervical cancer have a low quality of life and the stress caused by the disease, distorted sexual relationships, and lack of psychological acceptance (coping) make the disease course more challenging for these patients [19]. Additionally, prolonged course of treatment, along with the progression of the disease stage, are often accompanied by complications such as anemia, bleeding, etc., which can affect the survival rate of patients [20]. The advanced stage of the disease and their prolonged treatment have a significant psychological impact on their general health [21]. Moreover, radiotherapy and chemotherapy which are frequently used interventions for treatment of cervical cancer can result in sexual dysfunction, further reducing their quality of life [22]. Therefore, despite the mentioned problems, delayed diagnosis significantly decreases the survival rate of cervical cancer.

In a cohort study conducted in China, the time interval between diagnosis and treatment was investigated based on the tumor stage at the time of diagnosis, and cervical cancer is generally grouped into stages I, II, III, and IV. People whose time between diagnosis and treatment was more than 180 days had the worse prognosis, whereas those whose time between diagnosis and treatment was less than 90 days had lower mortality rate [23]. In a study conducted in Iranian in 2015, the late-stage diagnosis of cervical cancer was significantly higher in patients with low education, low socioeconomic status, those with a spouse who is a smoker or a drug addict, and those who did not have Pap smear screening tests according to the guidelines [24]. Poor access to information and screening resources, as well as the inequality of access to resources in rural areas compared to urban areas, lead to poor patient compliance; with the process of screening, diagnosis, treatment, and post-treatment [25]. Garner’s study published in 2022 shows that screening, diagnosis, and treatment processes are less noticeable in low-income countries (reference?). Nearly 90% of cervical cancer cases occur in low- and middle-income women in countries that lack organized HPV vaccination and screening programs; women with low income are usually diagnosed at advanced stages, resulting in a higher mortality rate. In high-income countries, the incidence and mortality rates from cervical cancer have been reduced by more than half since the implementation of screening programs [26]. The incidence and mortality rates of cervical cancer decrease with the increase of the Human Development Index (HDI) and the decrease of the Gender Inequality Index (GII). A two-tenth unit increase in HDI is associated with a 20% reduction in the risk of developing cervical cancer and a 33% reduction in cervical cancer mortality rate. For every 0.2 unit increase in the GII, the risk of cervical cancer diagnosis increases by 24% and death from cervical cancer increases by 42%, and higher levels of health expenditure are independently associated with lower incidence and mortality risks [27].

The results of the present meta-analysis study revealed that the survival of patients has increased with the increase of the study year towards more recent years, and this increase in survival is statistically significant. In agreement with the present study, it was elucidated that implementation of the screening program and promotion of vaccination, the disease could be detected in earlier stages [28]. In Asian countries, inhibiting factors that can prevent a patient from being diagnosed at an early stage can be a major contributor to late diagnosis and reduced life expectancy. Early diagnosis is frequently associated with patients’ knowledge of cervical cancer and also the overall level of their education [29] Therefore, despite vaccination and screening, preventing the progression of the disease to higher stages can influence the survival rate of patients.

The lowest and highest ten-year survival rates were reported in Taiwan and Kuwait, respectively. A population-based study conducted in Taiwan revealed that the prevalence of cervical cancer is still increasing [30]. Therefore, the role of risk factors and comorbid conditions such as diabetes mellitus, high blood pressure, smoking, having multiple sexual partners, etc., can be considered. The presence of these factors and conditions can have a negative impact on the survival rate of patients [31]. Another study revealed that half of the women in Kuwait were knowledgeable about cervical cancer, screening methods, and its risk factors. It was also determined that their willingness to repeat Pap smear tests is related to their career status, family income, and level of education [32]. Therefore, it is crucial to consider the level of awareness of the population about the disease, as well as the physical condition of the patient before treatment and the presence of comorbidities, which affects the survival rate.

The limitations of the present study are the type and quality of the studies that have been investigated in this study. The sample size of studies and the number of studies conducted in each country can impact the results as well for example, in the case of Kuwait and Iran. Additionally, more than half of the Asian countries had not published any studies on the survival of patients with cervical cancer; therefore, more studies are required to make a more accurate estimate, especially in countries without reports. Also, age information is essential for a more accurate analysis of survival rates. But due to lack of sufficient access to data, this aspect was not investigated as a separate factor in this study. It is suggested to pay more attention to this variable in future studies.

## Conclusion

Cervical cancer is highly preventable through Pap smear screening and vaccination. Additionally, by using Pap smear screening, the disease is diagnosed in early stages. The results of this study determined the survival rate in Asian countries and revealed that the ten-year survival rate of patients is low. This study fills a knowledge gap, and it is expected that by using the evidence-based data from this study, health intervention strategies and health policies can be developed to increase cervical cancer screening in order to improve preventive measures, and early diagnosis in Asian countries.

### Electronic supplementary material

Below is the link to the electronic supplementary material.

Supplementary Material 1

Supplementary Material 2

Supplementary Material 3

Supplementary Material 4

## Data Availability

The data that support the findings of this study are available from the corresponding author, [HGH], upon reasonable request.
