# Supplementary material for: Survival rate of cervical cancer in Asian countries: a systematic review and meta-analysis
Source: BMC Womens Health. 2023 Dec 14;23:671. doi: 10.1186/s12905-023-02829-8 (PMC10722657; doi:10.1186/s12905-023-02829-8)
Supplement: Supplementary file 1 — Supplementary Material 1 [file 12905_2023_2829_MOESM1_ESM.docx]

Appendix 1: **Search strategy**

**ISI / Proquest**

((cancer OR neoplasm OR Cervical Cancer OR Cervical Neoplasms OR Cervical Tumor OR Cancer of Cervical OR Neoplasms of Cervical OR Cervical carcinoma OR Cervix Cancer OR Cervix Neoplasm OR Cancer of the Cervix OR Cancer of Cervix) AND (Survival OR Survival Analysis OR Survival Rate) AND (Afghanistan OR Armenia OR Azerbaijan OR Bahrain OR Bangladesh OR Bhutan OR Brunei OR Myanmar OR Cambodia OR China OR Georgia OR Hong Kong OR India OR Indonesia OR Iran OR Iraq OR Japan OR Jordan OR Kazakhstan OR North Korea OR South Korea OR Kuwait OR Kyrgyzstan OR Laos OR Lebanon OR Macau OR Malaysia OR Maldives OR Mongolia OR Nepal OR Oman OR Pakistan OR Philippines OR Qatar OR Saudi Arabia OR Singapore OR Sri Lanka OR Syria OR Taiwan OR Tajikistan OR Thailand OR Timor-Leste OR Turkmenistan OR United Arab Emirates OR Uzbekistan OR Vietnam OR Yemen OR Asia OR Asian OR Asian countries OR Asian nation) NOT (Brain and nervous system OR Breast OR Endocrine system OR Gastrointestinal OR prostate OR colon OR colorectal OR Gastric OR stomach OR Pancreatic OR bladder OR esophageal OR oral OR Melanoma OR skin OR lung OR gynecological OR corpus uteri OR oesophageal OR larynx OR gallbladder OR rectum OR rectal OR blood OR lymphoma OR pediatric OR nasopharyngeal))

PubMed

(("Cervical Neoplasms"[Mesh]) AND (Survival[Title/Abstract] OR Survival Analysis[Title/Abstract] OR Survival Rate[Title/Abstract])) AND ((Afghanistan[Title/Abstract] OR Armenia[Title/Abstract] OR Azerbaijan[Title/Abstract] OR Bahrain[Title/Abstract] OR Bangladesh[Title/Abstract] OR Bhutan[Title/Abstract] OR Brunei[Title/Abstract] OR Myanmar[Title/Abstract] OR Cambodia[Title/Abstract] OR China[Title/Abstract] OR Georgia[Title/Abstract] OR Hong Kong[Title/Abstract] OR India[Title/Abstract] OR Indonesia[Title/Abstract] OR Iran[Title/Abstract] OR Iraq[Title/Abstract] OR Japan[Title/Abstract] OR Jordan[Title/Abstract] OR Kazakhstan[Title/Abstract] OR North Korea[Title/Abstract] OR South Korea[Title/Abstract] OR Kuwait[Title/Abstract] OR Kyrgyzstan[Title/Abstract] OR Laos[Title/Abstract] OR Lebanon[Title/Abstract] OR Macau[Title/Abstract] OR Malaysia[Title/Abstract] OR Maldives[Title/Abstract] OR Mongolia[Title/Abstract] OR Nepal[Title/Abstract] OR Oman[Title/Abstract] OR Pakistan[Title/Abstract] OR Philippines[Title/Abstract] OR Qatar[Title/Abstract] OR Saudi Arabia[Title/Abstract] OR Singapore[Title/Abstract] OR Sri Lanka[Title/Abstract] OR Syria[Title/Abstract] OR Taiwan[Title/Abstract] OR Tajikistan[Title/Abstract] OR Thailand[Title/Abstract] OR Timor-Leste[Title/Abstract] OR Turkmenistan[Title/Abstract] OR United Arab Emirates[Title/Abstract] OR Uzbekistan[Title/Abstract] OR Vietnam[Title/Abstract] OR Yemen[Title/Abstract] OR Asia[Title/Abstract] OR Asian[Title/Abstract] OR Asian countries[Title/Abstract] OR Asian nation)[Title/Abstract])

Scopus

TITLE-ABS (cancer OR neoplasm OR Cervical Cancer OR Cervical Neoplasms OR Cervical Tumor OR Cancer of Cervical OR Neoplasms of Cervical OR Cervical carcinoma OR Cervix Cancer OR Cervix Neoplasm OR Cancer of the Cervix OR Cancer of Cervix) AND TITLE-ABS (Survival OR Survival Analysis OR Survival Rate) AND TITLE-ABS() (Afghanistan OR Armenia OR Azerbaijan OR Bahrain OR Bangladesh OR Bhutan OR Brunei OR Myanmar OR Cambodia OR China OR Georgia OR Hong Kong OR India OR Indonesia OR Iran OR Iraq OR Japan OR Jordan OR Kazakhstan OR North Korea OR South Korea OR Kuwait OR Kyrgyzstan OR Laos OR Lebanon OR Macau OR Malaysia OR Maldives OR Mongolia OR Nepal OR Oman OR Pakistan OR Philippines OR Qatar OR Saudi Arabia OR Singapore OR Sri Lanka OR Syria OR Taiwan OR Tajikistan OR Thailand OR Timor-Leste OR Turkmenistan OR United Arab Emirates OR Uzbekistan OR Vietnam OR Yemen OR Asia OR Asian OR Asian countries OR Asian nation)
