# Supplementary material for: Survival rate of cervical cancer in Asian countries: a systematic review and meta-analysis
Source: BMC Womens Health. 2023 Dec 14;23:671. doi: 10.1186/s12905-023-02829-8 (PMC10722657; doi:10.1186/s12905-023-02829-8)
Supplement: Supplementary file 2 — Supplementary Material 2 [file 12905_2023_2829_MOESM2_ESM.docx]

Newcastle-Ottawa Quality Assessment Form for Cohort Studies

| Quality^*^ | Total | Outcome | Comparability | Selection | Author (year) |
| --- | --- | --- | --- | --- | --- |
| Good | 7 | 3 | 2 | 2 | Al Asiri M et al.2013 |
| Good | 7 | 3 | 1 | 3 | Abdreza N.2013 |
| Good | 7 | 3 | 1 | 3 | Abu-Zaid A.2017 |
| Fair | 6 | 3 | 1 | 2 | Akahira, J.2006 |
| Fair | 6 | 3 | 1 | 2 | Aleyamma Mathew,2020 |
| Fair | 6 | 3 | 1 | 2 | Angeline Gnanamalar,2019 |
| Good | 7 | 3 | 1 | 3 | Aoki D.2014 |
| Fair | 6 | 3 | 1 | 2 | Arimoto T.1991 |
| Good | 7 | 3 | 1 | 3 | Asami Yagi,2019 |
| Fair | 6 | 3 | 1 | 2 | Ayhan A.2006 |
| Good | 6 | 2 | 1 | 3 | Balasubramaniam, G.2013 |
| Good | 7 | 3 | 1 | 3 | Basu P.2006 |
| Good | 7 | 3 | 1 | 3 | Bates,G.H.2008 |
| Fair | 6 | 3 | 1 | 2 | Behtash N.2009 |
| Good | 6 | 2 | 1 | 3 | Bhika B.2004 |
| Good | 7 | 3 | 1 | 3 | Binesh, F.2014 |
| Good | 7 | 3 | 1 | 3 | Biswal B.M.1994 |
| Good | 7 | 3 | 1 | 3 | Boupaijit, K.2016 |
| Fair | 6 | 3 | 1 | 2 | Cai, H.B.2006 |
| Good | 6 | 2 | 1 | 3 | Chang S.J.2008 |
| Fair | 6 | 3 | 1 | 2 | Chen C.Y.2012 |
| Good | 6 | 2 | 1 | 3 | Chen J.R.2016 |
| Fair | 6 | 3 | 1 | 2 | Chen L.2010 |
| Good | 6 | 2 | 1 | 3 | Chen M.2015 |
| Good | 7 | 3 | 1 | 3 | Chen M. S.1990 |
| Good | 7 | 3 | 1 | 3 | Chen R.J.1998 |
| Fair | 6 | 3 | 1 | 2 | Chen, J. G. 1998 |
| Good | 7 | 3 | 1 | 3 | Chen, J. G. 2011 |
| Good | 6 | 2 | 1 | 3 | Chen, J. G.2018 |
| Fair | 6 | 3 | 1 | 2 | Chen, L.2010 |
| Good | 6 | 2 | 1 | 3 | Chen, M.2015 |
| Good | 7 | 3 | 1 | 3 | Chen,C.C.2012 |
| Good | 7 | 3 | 1 | 3 | Chen,J.G.2018 |
| Fair | 6 | 3 | 1 | 2 | Chen,J.L.Y.2012 |
| Good | 7 | 3 | 1 | 3 | Chen,J.R.2016 |
| Fair | 6 | 3 | 1 | 2 | Chen,T.H.2017 |
| Fair | 6 | 3 | 1 | 2 | Chen.L.2010 |
| Good | 6 | 2 | 1 | 3 | Cheng, X. 2004 |
| Good | 6 | 2 | 1 | 3 | Cheung,F.2011 |
| Good | 7 | 3 | 1 | 3 | Cheung,FY.2011 |
| Good | 6 | 2 | 1 | 3 | Chia-Hao Liu,2019 |
| Good | 7 | 3 | 1 | 3 | Chia, K.S.2011 |
| Good | 7 | 3 | 1 | 3 | Chia,K.S.2001 |
| Good | 7 | 3 | 1 | 3 | Chiou,W.Y.2016 |
| Good | 7 | 3 | 1 | 3 | Cho,H.2014 |
| Fair | 6 | 3 | 1 | 2 | Choi,P.1992 |
| Good | 7 | 3 | 1 | 3 | Chu,k.k.1998 |
| Fair | 6 | 3 | 1 | 2 | Chung.H.H.2006 |
| Fair | 6 | 3 | 1 | 2 | Chunlin Chen,2019 |
| Good | 6 | 2 | 1 | 3 | Chunlin Chen,2020 |
| Fair | 5 | 2 | 1 | 2 | Corazon A.2001 |
| Fair | 6 | 3 | 1 | 2 | Cuylan,Z.F.2018 |
| Fair | 5 | 2 | 1 | 2 | Khosla D.2012 |
| Fair | 6 | 3 | 1 | 2 | Dahiya,N.2016 |
| Fair | 6 | 3 | 1 | 2 | Dan Li,2019 |
| Good | 6 | 2 | 1 | 3 | Barmon D,2020 |
| Fair | 6 | 3 | 1 | 2 | Dikshit, R, 2011 |
| Good | 7 | 3 | 1 | 3 | Ding-Ding Yan,2019 |
| Good | 7 | 3 | 1 | 3 | Dipak Jagdishchandra Limbachiya,2020 |
| Good | 7 | 3 | 1 | 3 | Dong hyun kim.2016 |
| Good | 7 | 3 | 1 | 3 | Dong Wook Shin,2020 |
| Good | 7 | 3 | 1 | 3 | E EL SAYED,M.2016 |
| Fair | 6 | 3 | 1 | 2 | Alawadhi E,2019 |
| Good | 6 | 2 | 1 | 3 | EL-SENOUSSI.M.1998 |
| Good | 6 | 2 | 1 | 3 | Elantholi P.2005 |
| Good | 6 | 2 | 1 | 3 | Erdem,O.2006 |
| Good | 6 | 2 | 1 | 3 | Eric J.S.2005 |
| Good | 6 | 2 | 1 | 3 | Esteban, D.1998 |
| Good | 7 | 3 | 1 | 3 | Fei Cao,2019 |
| Fair | 6 | 3 | 1 | 2 | Feng,W.2011 |
| Good | 7 | 3 | 1 | 3 | Fujimoto,T.2007 |
| Fair | 6 | 3 | 1 | 2 | Gek-Hsiang Lim.2009 |
| Good | 7 | 3 | 1 | 3 | Genara A.1997 |
| Good | 7 | 3 | 1 | 3 | Ghosh, S.2015 |
| Good | 7 | 3 | 1 | 3 | Ghosh, S.2016 |
| Good | 7 | 3 | 1 | 3 | Gong, L.2011 |
| Good | 7 | 3 | 1 | 3 | Goto, T.2005 |
| Good | 7 | 3 | 1 | 3 | Goura K.2010 |
| Good | 7 | 3 | 1 | 3 | Gue,J.2018 |
| Good | 7 | 3 | 1 | 3 | H-J. Huang.2003 |
| Fair | 6 | 3 | 1 | 2 | Khalkhali HR,2019 |
| Good | 7 | 3 | 1 | 3 | Hongo, A.2010 |
| Good | 7 | 3 | 1 | 3 | Ikushima, H.2007 |
| Good | 7 | 3 | 1 | 3 | Inoue S.2018 |
| Good | 7 | 3 | 1 | 3 | Jayalekshmi, P.2011 |
| Good | 7 | 3 | 1 | 3 | Jayant K.1996 |
| Good | 7 | 3 | 1 | 3 | Jayant, K.2011 |
| Fair | 6 | 3 | 1 | 2 | Jin, F. 1998 |
| Good | 7 | 3 | 1 | 3 | Kei Ito,2018 |
| Good | 7 | 3 | 1 | 3 | Laudico, A.Mapua, C.2011 |
| Good | 7 | 3 | 1 | 3 | Law, S. C.2011 |
| Good | 7 | 3 | 1 | 3 | Martin, N.2011 |
| Good | 7 | 3 | 1 | 3 | Myong cheol Lim.2018 |
| Good | 7 | 3 | 1 | 3 | Nandakumar A.1993 |
| Good | 7 | 3 | 1 | 3 | Pesee M.2013 |
| Good | 7 | 3 | 1 | 3 | Phanphaisarn.2016 |
| Good | 7 | 3 | 1 | 3 | Rittiluechai K.2010 |
| Good | 7 | 3 | 1 | 3 | Shanta, V. 1998 |
| Good | 7 | 3 | 1 | 3 | Shen S.C.2016 |
| Good | 7 | 3 | 1 | 3 | Shusaku Inoue.2019 |
| Good | 7 | 3 | 1 | 3 | Sriamporn, S.1995 |
| Good | 7 | 3 | 1 | 3 | Sriplung, H.2011 |
| Fair | 6 | 3 | 1 | 2 | Sumitsawan, Y.2011 |
| Good | 6 | 2 | 1 | 3 | Vatanasapt, V.1998 |
| Good | 6 | 2 | 1 | 3 | Xiang, Y. B.2011 |
| Good | 6 | 2 | 1 | 3 | Y.Kuwabara.2005 |
| Good | 6 | 2 | 1 | 3 | Yan X.2011 |
| Good | 6 | 2 | 1 | 3 | Yeole, B. B.2011 |
| Good | 7 | 3 | 1 | 3 | Yevgeniy S.2015 |
| Fair | 6 | 3 | 1 | 2 | Zhu J.H.2018 |

* **Newcastle-Ottawa Quality Assessment Form for Cohort Studies**
