# Supplementary material for: Survival rate of cervical cancer in Asian countries: a systematic review and meta-analysis
Source: BMC Womens Health. 2023 Dec 14;23:671. doi: 10.1186/s12905-023-02829-8 (PMC10722657; doi:10.1186/s12905-023-02829-8)
Supplement: Supplementary file 3 — Supplementary Material 3 [file 12905_2023_2829_MOESM3_ESM.docx]

Appendix 2: Basic information of Included Studies

| **Order** | **Author (year)** | **location** | **Time period** | **Sample size** | **cervical**  **Survival Rate** | | | |
| --- | --- | --- | --- | --- | --- | --- | --- | --- |
|  |  |  |  |  | **1** | **3** | **5** | **10** |
| 1 | Al Asiri M et al.2013 | Saudi Arabia | 2007-2012 | 45 | 76.00 | 65.00 | 62.00 | - |
| 2 | Abdreza N.2013 | Malaysia | 1995-2007 | 120 | 35.00 | 41.00 | 55.00 | 45.00 |
| 3 | Abu-Zaid A.2017 | Saudi Arabia | 2010-2013 | 162 | - | - | 50.00 | - |
| 4 | Akahira, J.2006 | Japan | 1990-2004 | 921 | 52 | 61 | 68 | - |
| 5 | Aleyamma Mathew,2020 | India | 2012-2014 | 364 | - | - | 51.00 | - |
| 6 | Angeline Gnanamalar,2019 | India | 2001-2010 | 61 | 89.00 | 84.00 | 79.00 | - |
| 7 | Aoki D.2014 | Japan | 2005-2011 | 156 | - | - | 91.00 | - |
| 8 | Arimoto T.1991 | Japan | 1983-1987 | 15 | 92.00 | 93.00 | 90.00 | - |
| 9 | Asami Yagi,2019 | Japan | 1976-2012 | 503 | 64.00 | 60.00 | - | - |
| 10 | Ayhan A.2006 | Turkey | 1974-2005 | 50 | 85.00 | 89.00 | 88.00 | 80.00 |
| 11 | Balasubramaniam, G.2013 | India | 1999-2002 | 310 | 92.00 | 93.00 | - | - |
| 12 | Basu P.2006 | India | 1999 | 194 | 29.00 | 44.00 | 54.00 | 59.00 |
| 13 | Bates,G.H.2008 | Philippines | 1990-2004 | 805 | 79.00 | 75.00 | - | - |
| 14 | Behtash N.2009 | Iran | 1995-2002 | 203 | 81.00 | 83.00 | - | - |
| 15 | Bhika B.2004 | India | 1992-1994 | 486 | 56.00 | 68.00 | 41.00 | 48.00 |
| 16 | Binesh, F.2014 | Iran | 2005-2012 | 84 | - | - | 78.00 | - |
| 17 | Biswal B.M.1994 | India | 1987-1988 | 271 | - | - | 65.00 | - |
| 18 | Boupaijit, K.2016 | Thailand | 2008-2014 | 173 | - | - | 15.00 | - |
| 19 | Cai, H.B.2006 | China | 1999-2002 | 165 | - | - | 80.00 | - |
| 20 | Chang S.J.2008 | Korea | 1994-2007 | 160 | 81.00 | 86.00 | 80.00 | - |
| 21 | Chen C.Y.2012 | Taiwan | 1979-2008 | 529 | 18.00 | 25.00 | 65.00 | - |
| 22 | Chen J.R.2016 | Taiwan | 1991-2010 | 541 | - | - | 70.00 | - |
| 23 | Chen L.2010 | China | 1997-2009 | 818 | 82.00 | 75.00 | - | - |
| 24 | Chen M.2015 | China | 2000-2011 | 33 | - | 71.00 | - | - |
| 25 | Chen M. S.1990 | Taiwan | 1980-1985 | 399 | 42.00 | 43.00 | 53.00 | - |
| 26 | Chen R.J.1998 | Taiwan | 1977-1994 | 369 | - | 29.00 | 63.00 | 26.00 |
| 27 | Chen, J. G. 1998 | China | 1982-1991 | 173 | 37.00 | 29.00 | - | 26.00 |
| 28 | Chen, J. G. 2011 | China | 1992-2000 | 144 | 39.00 | 37.00 | 46.00 | - |
| 29 | Chen, J. G.2018 | China | 2002-2014 | 501 | - | - | 58.00 | - |
| 30 | Chen, L.2010 | China | 1997-2009 | 18 | 82 | 75 | - | - |
| 31 | Chen, M.2015 | China | 2000-2011 | 33 | - | - | 71.00 | - |
| 32 | Chen,C.C.2012 | Taiwan | 2004-2010 | 125 | - | - | 74.00 | - |
| 33 | Chen,J.G.2018 | China | 2002-2014 | 501 | 73 | 67 | 58.00 | - |
| 34 | Chen,J.L.Y.2012 | Taiwan | 1995-2009 | 170 | - | - | 67.00 | - |
| 35 | Chen,J.R.2016 | Taiwan | 1991-2010 | 541 | - | - | 70.00 | - |
| 36 | Chen,T.H.2017 | Taiwan | 1993-2014 | 562 | - | - | 68.00 | - |
| 37 | Chen.L.2010 | China | 1997-2009 | 18 | 82.00 | - | 75.00 | - |
| 38 | Cheng, X. 2004 | China | 1992-1997 | 19 | 49.00 | 57.00 | 54.00 | 56.00 |
| 39 | Cheung,F.2011 | Hong Kong | 1997-2006 | 380 | 91.00 | 77.00 | 71.00 | 73.00 |
| 40 | Cheung,FY.2011 | Hong Kong | 1997-2006 | 296 | 80.00 | 64.00 | 60.00 | - |
| 41 | Chia-Hao Liu,2019 | Taiwan | 2012-2014 | 39 | - | 54.00 | 43.00 | - |
| 42 | Chia, K.S.2011 | Singapore | 1968-1997 | 984 | 84.00 | 66.00 | 60.00 | - |
| 43 | Chia,K.S.2001 | Singapore | 1988–1992 | 901 | 63 | 56 | 48 | - |
| 44 | Chiou,W.Y.2016 | Taiwan | 2000-2010 | 244 | - | - | 95.00 | - |
| 45 | Cho,H.2014 | Korea | 2006-2008 | 48 | - | 88.00 | 83.00 | - |
| 46 | Choi,P.1992 | Hong Kong | 1984-1988 | 136 | - | - | 63.00 | - |
| 47 | Chu,k.k.1998 | Taiwan | 1992-1995 | 43 | 86.00 | - | - | - |
| 48 | Chung.H.H.2006 | Korea | 1993–2002 | 699 | 99.00 | 97.00 | 96.00 | 94.00 |
| 49 | Chunlin Chen,2019 | China | 2004-2016 | 200 | - | 90 | 85 | - |
| 50 | Chunlin Chen,2020 | China | 2009-2016 | 198 | - | - | - | - |
| 51 | Corazon A.2001 | Philippines | 1995-1998 | 152 | - | - | 27.00 | - |
| 52 | Cuylan,Z.F.2018 | Turkey | 2001-2016 | 172 | - | - | 75.00 | - |
| 53 | Khosla D.2012 | India | 2005-2010 | 252 | 76 | 74 | 67 | - |
| 54 | Dahiya,N.2016 | India | 2014-2014 | 67 | - | - | 93.00 | - |
| 55 | Dan Li,2019 | China | 2008-2013 | 1435 | - | - | 94.00 | - |
| 56 | Barmon D,2020 | India | 2014-2015 | 38 | - | - | 92.00 | - |
| 57 | Dikshit, R, 2011 | India | 1991-1995 | 332 | 89.00 | 66.00 | - | - |
| 58 | Ding-Ding Yan,2019 | China | 2008-2011 | 155 | 91.00 | 81.00 | 79.00 | - |
| 59 | Dipak Jagdishchandra Limbachiya,2020 | India | 2012-2017 | 88 | 95.00 | - | - | - |
| 60 | Dong hyun kim.2016 | Korea | 2007-2012 | 116 | 70.00 | 70.00 | 43.00 | - |
| 61 | Dong Wook Shin,2020 | Korea | 1996–2015 | 786 | 81.00 | - | - | - |
| 62 | E EL SAYED,M.2016 | Saudi Arabia | 2004-2010 | 60 | - | 82.00 | 79.00 | - |
| 63 | Alawadhi E,2019 | Kuwait | 2010-2013 | 163 | 86.00 | 84.00 | 74.00 | 89.00 |
| 64 | EL-SENOUSSI.M.1998 | Saudi Arabia | 1979-1991 | 164 | 86.00 | 94.00 | 75.00 | - |
| 65 | Elantholi P.2005 | India | 1996-2001 | 105 | 86.00 | 79.00 | 70.00 | 50.00 |
| 66 | Erdem,O.2006 | Turkey | 1990-2003 | 90 | - | 97.00 | 76.00 | - |
| 67 | Eric J.S.2005 | Vietnam | 1996-1996 | 224 | 63.00 | 73.00 | 71.00 | - |
| 68 | Esteban, D.1998 | Philippines | 1983-1987 | 192 | 69.00 | 37.00 | 27.00 | - |
| 69 | Fei Cao,2019 | China | 2012-2018 | 89 | 75.00 | 64.00 | - | - |
| 70 | Feng,W.2011 | China | 1954-2007 | 68 | - | - | - | 93.00 |
| 71 | Fujimoto,T.2007 | Japan | 1993-2004 | 21 | - | 60.00 | - | 19.00 |
| 72 | Gek-Hsiang Lim.2009 | Singapore | 1998-2002 | 1422 | 64 | 60 | 42 | - |
| 73 | Genara A.1997 | Philippines | 1961-1990 | 120 | - | 47.00 | 48.00 |  |
| 74 | Ghosh, S.2015 | India | 2012-2014 | 76 |  | 82.00 | - | - |
| 75 | Ghosh, S.2016 | India | 2012-2013 | 124 | 93.00 | - | - | - |
| 76 | Gong, L.2011 | China | 2008-2009 | 414 | - | 96.00 | 93.00 | - |
| 77 | Goto, T.2005 | Japan | 1977-1721 | 172 | - | 51.00 | 22.00 | - |
| 78 | Goura K.2010 | India | 2003-2005 | 48 | 82.00 | 80.00 | 78.00 | - |
| 79 | Gue,J.2018 | China | 2003-2016 | 143 | - | - | 98.00 | - |
| 80 | H-J. Huang.2003 | Taiwan | 1991-2010 | 157 | 77.00 | 72.00 | 42.00 | - |
| 81 | Khalkhali HR,2019 | Iran | 2004-2015 | 109 | 99.00 | - | - | - |
| 82 | Hongo, A.2010 | Japan | 2001-2006 | 39 | 95.00 | - | - | - |
| 83 | Ikushima, H.2007 | Japan | 1969-1997 | 727 | - | - | - | 30.00 |
| 84 | Inoue S.2018 | Japan | 2001-2006 | 119 | 73.00 | - | - | - |
| 85 | Jayalekshmi, P.2011 | India | 1991-1997 | 170 | 83.00 | - | - | - |
| 86 | Jayant K.1996 | India | 1990-1991 | 111 | 64.00 | - | - | - |
| 87 | Jayant, K.2011 | India | 1993-2000 | 406 | 71.00 | - | - | - |
| 88 | Jin, F. 1998 | China | 1988-1991 | 619 | 74.00 | - | - | - |
| 89 | Kei Ito,2018 | Japan | 2005-2017 | 327 | 74.00 | - | - | - |
| 90 | Laudico, A.Mapua, C.2011 | Philippines | 1994-1995 | 377 | 64.00 | - | - | - |
| 91 | Law, S. C.2011 | China | 1996-2001 | 262 | 89.00 | - | - | - |
| 92 | Martin, N.2011 | Thailand | 1990-2000 | 107 | 86.00 | - | - | - |
| 93 | Myong cheol Lim.2018 | Korea | 1993-2011 | 147 | - | - | - | 80.00 |
| 94 | Nandakumar A.1993 | India | 1988-1992 | 100 | 76.00 | - | - | - |
| 95 | Pesee M.2013 | Thailand | 2003-2005 | 30 | 88.00 | - | - | - |
| 96 | Phanphaisarn.2016 | Thailand | 2006-2015 | 294 | 47.00 | - | - | - |
| 97 | Rittiluechai K.2010 | Thailand | 1991-2006 | 229 | 67.00 | - | - | - |
| 98 | Shanta, V. 1998 | India | 1984-1989 | 328 | 88.00 | - | - | - |
| 99 | Shen S.C.2016 | Taiwan | 2005-2010 | 908 | 91.00 | - | - | - |
| 100 | Shusaku Inoue.2019 | Japan | 2006-2008 | 1309 | 78.00 | - | - | - |
| 101 | Sriamporn, S.1995 | Thailand | 1985-1992 | 857 | 86.00 | - | - | - |
| 102 | Sriplung, H.2011 | Thailand | 1990-1999 | 780 | 89.00 | - | - | - |
| 103 | Sumitsawan, Y.2011 | Thailand | 1993–1997 | 885 | 86.00 | - | - | - |
| 104 | Vatanasapt, V.1998 | Thailand | 1985-1992 | 820 | 85.00 | - | - | - |
| 105 | Xiang, Y. B.2011 | China | 1992-1995 | 548 | 77.00 | - | - | - |
| 106 | Y.Kuwabara.2005 | Japan | 1975-2002 | 890 | - | - | - | 95.00 |
| 107 | Yan X.2011 | China | 2000-2010 | 148 | 91.00 | - | - | - |
| 108 | Yeole, B. B.2011 | India | 1992-1994 | 443 | 75.00 | - | - | - |
| 109 | Yevgeniy S.2015 | Japan | 1997-2012 | 139 | - | - | - | 81.00 |
| 110 | Zhu J.H.2018 | China | 2012-2016 | 28 | 99.00 | - | - | - |
