# Supplementary material for: Survival rate of cervical cancer in Asian countries: a systematic review and meta-analysis
Source: BMC Womens Health. 2023 Dec 14;23:671. doi: 10.1186/s12905-023-02829-8 (PMC10722657; doi:10.1186/s12905-023-02829-8)
Supplement: Supplementary file 4 — Supplementary Material 4 [file 12905_2023_2829_MOESM4_ESM.docx]

|   (A) | **(B)** |
| --- | --- |
|   (C ) |   (D) |

**Appendix 4:** Funnel plot of standard error by point estimate for assessment of publication bias (one, 3, five and ten year Cervix cancer survival rate)[A: One Cervix cancer survival rate, B: three Cervix cancer survival rate, C:Five Cervix cancer survival rate, D: Ten Cervix cancer survival rate]
